# Paraneoplastic Pemphigus Autoantibodies Against C-terminus of Desmoplakin Induced Acantholysis *In Vitro* and *In Vivo*

**DOI:** 10.3389/fimmu.2022.886226

**Published:** 2022-07-14

**Authors:** Xue Wang, Rui Wang, Dingfang Bu, Leyi Wang, Yuexin Zhang, Yuan Chang, Chenyang Zhang, Xixue Chen, Xuejun Zhu, Zhi Liu, Mingyue Wang

**Affiliations:** ^1^ Department of Dermatology, Peking University First Hospital, Beijing, China; ^2^ National Clinical Research Center for Skin and Immune Diseases, Beijing, China; ^3^ Beijing Key Laboratory of Molecular Diagnosis on Dermatoses, Beijing, China; ^4^ Department of Dermatology, Beijing Children's Hospital, Capital Medical University, National Center for Children's Health, Beijing, China; ^5^ Central Laboratory, Peking University First Hospital, Beijing, China; ^6^ Department of Dermatology, Central Hospital, Zhengzhou University, Zhengzhou, China; ^7^ Department of Dermatology, University of North Carolina, Chapel Hill, NC, United States

**Keywords:** Paraneoplastic pemphigus, desmoplakin, plakin, desmoglein, acantholoysis, mouse model

## Abstract

Paraneoplastic pemphigus (PNP) is an autoimmune bullous disease associated with underlying neoplasms and characterized by antibodies against desmoglein 3 (Dsg 3) and plakins. Autoantibodies against desmoglein 3 in sera of patients with PNP have been proven to cause acantholysis *in vivo* in neonatal mice. As a member of the plakin family, autoantibodies against desmoplakin were detected frequently by immunoprecipitation in the sera of PNP. The recombinant C-terminus of desmoplakin was expressed and purified to adsorb the specific autoantibodies against the C-terminus of desmoplakin. *In vitro* dispase-dependent keratinocyte dissociation assay and *in vivo* IgG passive transfer into neonatal mice assay were performed, followed by the electronic microscopy examination and TUNEL assay. We found that anti-C terminus of desmoplakin autoantibodies caused blisters and acantholysis in mice skin at a dose-dependent manner. Moreover, dissociated fragments were observed after incubation with the purified IgG against desmoplakin, compared with normal human IgG (*P*-value =0.0207). The electronic microscopy examination showed the disconnection of keratin intermediate filaments from desmosomes. Lastly, apoptosis of keratinocytes in the TUNEL assay was all detected in the skins of neonatal mice after injection of the anti-C terminus of desmoplakin autoantibodies. Taken together, the study suggests that autoantibodies against the C-terminus of desmoplakin might be pathogenic in PNP.

## Introduction

Paraneoplastic pemphigus (PNP) is a multi-organ syndrome with multiple autoantibodies (1). Besides desmoglein 1, desmoglein 3, and α-2-macroglobulin-like-1 protein, several members of the plakin family such as epiplakin, plectin, desmoplakin (DP), bullous pemphigoid antigen 1, envoplakin, and periplakin are particularly found to be the antigens of PNP. In the past decades, we investigated the role of the plakin family in PNP and proved that tumor cells of Castleman disease with PNP could secrete antibodies against plakin family proteins and cause detachment of cultured keratinocytes (2). Followed by epitope-mapping, we discovered that the extremities of the N-terminus of envoplakin and the C-terminus of its linker subdomain are major epitopes of PNP (3). However, patients’ antibodies purified by these two proteins failed to cause any pathological changes in animal models. So far, the animal model has demonstrated the pathogenetic role of anti-desmoglein 3 antibodies (4), but it cannot explain the absence or low levels of anti-desmoglein 3 antibodies in a significant portion of PNP patients.

Among the plakin family, DP, most frequently detected by immunoprecipitation (IP), shares high homology with the others, especially envoplakin and periplakin (5), which were almost universally detected by immunoblotting (IB). However, the pathogenic role of autoantibodies against DP in PNP remained to be investigated (6). Interestingly, autoantibodies against desmoplakin were occasionally detected in erythema multiforme major patients (7, 8). Autoantibodies against the peptide (GNSSYSYSYSFS) of the desmoplakin C-terminus in erythema multiforme major patients or sera of peptide-immuned rabbits have been shown to cause dyskeratosis and suprabasal acantholysis (9). Moreover, desmoplakin mutations can cause hereditary diseases such as lethal acantholytic epidermolysis bullousa (LAEB) (10). Based on the shared phenotypes of PNP and erythema multiforme and LAEB, we speculated that the autoantibodies against desmoplakin in PNP could contribute to the pathogenesis and cause at least some clinical phenotypes in PNP.

## Materials and Methods

### PNP Patient and Sera

Serum was obtained by plasmapheresis from a 16-year-old female patient diagnosed as PNP. The criteria we applied included progressive stomatitis, histologic features of acantholysis or lichenoid or interface dermatitis, demonstration of serum antiplakin autoantibodies by immunoblotting or immunoprecipitation, and the presence of an underlying lymphoproliferative neoplasm (11).

This patient presented with painful mucosal and mucocutanous ulcetrations for 4 months before admission. Severe painful erosions involved her tongue, lips, buccal mucosa, eyes, and vulva diffusely. General exfoliative skins were noted on her body. The direct immunofluorescence (DIF) showed deposition of C3 on the basement membrane zone, while indirect immunofluorescence (IIF) showed deposition of IgG on the surface of keratinocytes with the titer at 1:640. IIF using rat bladder as substrate was also positive and the titer was 1:160. IP combined IB detected antibodies against desmoplakin, envoplakin, periplakin, and desmoglein 3. A mass of 5 cm plus 4 cm in the mediastinum was detected by computed tomography. She was diagnosed with PNP and treated with plasmapheresis. Unfortunately, while preparing for the operative status, the patient died of sepsis.

### Expression of Recombinant C-Terminal of Desmoplakin

Full-length cDNA fragments encoding human desmoplakin I were synthesized and the target gene of the C-terminal (aa1945-aa2871) of desmoplakin (DP-C) was optimized and cloned into pET-28a (+) by NdeI and XhoI commercially (Genscript, Nanjing, China). The gene of C-terminal of desmoplakin (*DP-C*) with a C-terminal hexahistidine tag was transformed and expressed in *E. coli* BL21 (DE3). Briefly, a fresh BL21 (DE3) colony of 3 ml LB medium with 50µg/ml ampicillin was inoculated with 300 ml of LB medium supplemented with 50 μg/ml kanamycin. The LB medium was incubated at 37°C, shaking at 200 rpm until the bacterial suspension reached an optical density (OD) of 0.5 at 600 nm. After 5 h of induction with 0.05 mM/ml isopropyl-b-D-thiogalactoside, the culture was centrifuged. Soluble recombinant DP-C was obtained by sonication and purified by the NTA column (GE, Novagen) with binding buffer, washing buffer, and elution buffer as before (3). Imidazole was removed from the eluted protein by dialysis for 2-3 times at 4°C with phosphate buffered saline (PBS). Then recombinant DP-C was ultrafiltered to concentration and was measured by bicinchoninic acid assay. Finally, sodium dodecylsulfate-polyacrylamide gel electrophoresis (SDS-PAGE) was performed and stained with Coomassie brilliant blue.

### Recombinant DP-C was Coupled to CNBr-activated Sepharose 4B Beads for Preparation of the Affinity Column

A certain amount of CNBr-activated Sepharose 4B beads (GE Healthcare) was weighed and suspended in 1 mM HCl. The medium was washed for 15 min with 1 mM HCl following the protocol described in the instruction. Purified DP-C proteins were dialyzed against coupling buffer (0.1 M NaHCO3, 0.5 M NaCl, pH 8.3) and coupled with the medium overnight at 4°C. After washing away excess ligands with at least five times the medium volume of coupling buffer, we transferred the medium to 0.1 M Tris-HCl buffer (pH 8.0) to block any remaining active groups for 2 h, and then washed the medium with three cycles of alternating pH with five medium volumes of each buffer. Each cycle consisted of a wash with 0.1 M acetic acid/sodium acetate, pH 4.0 containing 0.5 M NaCl, followed by a wash with 0.1 M Tris-HCl, pH 8.0 containing 0.5 M NaCl.

### HPLC Affinity Purification

Firstly, normal adults’ sera and PNP patients’ sera were diluted with 0.02 M PBS and purified by rProteinA sepharose (GE Healthcare) on AKTA to obtain total IgG. The autoantibodies against DP-C were purified by the AKTA using the recombinant DP-C coupled to the CNBr column. IgG bound to DP-C was eluted (100 mM Glycine, pH 2.7) from the DP-C coupled sepharose columns on AKTA and neutralized with 2 M Tris-HCl, pH 8.0, and then it was concentrated by ultrafiltration with a 0.22 μm filter (Amicon.Millipore, Ireland) against PBS. The IgG of the N-terminus of desmoplakin was purified by the N-terminus of DP and followed the same strategy as the anti-DP-C IgG from the remaining IgG of the same patient after the DP-C affinity chromatography.

### Combined IP-IB

IP combined IB assay was performed using HaCat cells extract as substrate. The HaCat lysate buffer contained 62.5 mM Tris-buffer (PH = 8.0) with 1% TritonX-100 and the protease inhibitor cocktail tablet (Roche). The HaCat cells extract was precleared with rProtein A Sepharose by incubating it for 45 min at 4°C. Then 25 μg of purified DP-C specific IgG or positive controls (the commercial monoclonal antibodies against DP: Santa Cruz, sc-390975; the commercial monoclonal anti-desmoglein3 IgG: Abcam, ab14416) or healthy donors’ IgG was added to the precleared lysate separately and incubated overnight at 4°C, and then immunoprecipitated with rProtein A Sepharose for 2 h. The immunoprecipitants were washed for six times and separated by SDS-PAGE with a 6% gel, and electrotransferred onto nitrocellulose (NC) membranes. Then, after Ponceau S stained the NC membrane, only the wide and deeply dyed band of 55KDa was visible, most probably corresponding to the Ig heavy chains. In the following IB assay, desmoplakin and desmoglein 3 were detected by other commercial monoclonal antibodies (the monoclonal antibodies against desmoplakin I/II: Abcam, ab247866; the monoclonal antibodies against desmoglein 3: Abcam, ab128927).

### ELISA

The Desmoglein 3 ELISA kit (MBL, Japan) was used to confirm whether the purified IgG contained the desmoglein3-specific IgG. Normal control IgGs from six healthy volunteers, positive control IgGs from five PNP patients, and negative control from one PNP patient were included to determine the proper dilution ratio. IgG purified by rProteinA was diluted at 1 mg/ml and the assay was performed afterward. The proper dilution ratio of the IgG was determined when the cut-off value (mean + 3SD) could distinguish between positive and negative control. IgG was diluted by dilution buffer in the kit. The proper diluted ratio was 1:15. Other steps were strictly performed as per the manufacturer’s instructions.

### Dispase-Based Dissociation Assay

HEK (PromoCell) cells were seeded in triplicate on 12-well plates containing 0.1 mM calcium, then switched to the medium containing 1.2 mM calcium after reaching confluence, as described by Saleh et al. Cells were incubated in the medium containing 80 μg/ml of purified anti-DP-C autoantibodies or normal human IgG at 37°C overnight. Following washing in PBS, cells were incubated *in situ* with 0.3 ml of dispase II (> 2.4 U/ml; Roche) for 30 min to detach the epidermal sheet from the wells. The released sheets were carefully washed in PBS and then subjected to mechanical stress by pipetting with a 1 mL pipette. Fragments were fixed by adding formaldehyde at a final concentration of 3% and stained by adding crystal violet (Sigma-Aldrich) (12). At least three independent experiments were done in duplicates for each autoantibody.

### Passive Transfer of the Purified IgG

The concentration of the IgG was calculated by measuring OD280 (OD280 of 1 mg/ml IgG =1.43). IgG was affinity-purified on a recombinant DP-C column. Neonatal BALB/C mice (Beijing Vital River Laboratory Animal Technology, China) were obtained at 12–24 h of age (body weight, 1.4–1.6 g). The affinity-purified anti-DP-C IgG was injected at 3.3, 1.7, and 0.6 mg of total protein per gram of body weight into the subcutaneous of neonatal mice. Normal IgG was injected as a control with the same dose. Parts of the neonatal mice were sacrificed at 3 h and skins were harvested for direct immunofluorescence by freezing in optimal cutting temperature compound (OCT). The other mice were sacrificed at 24 h and skins were also obtained for HE staining, DIF, TUNEL assay, by either fixation in 10% formalin, freezing in OCT, or putting in the glutaraldehyde fixed solution prepared for electronic microscopy accordingly.

### Direct Immunofluorescence (DIF)

Neonatal mice skins used as substrate for DIF were biopsied 3 h after injection. The mice skins were embedded in OCT and made frozen sections with a thickness of 4-5 um. Fixed with acetone at 4°C for 10 min, and immersed with 0.01 mol/L PBS (pH7.4) in 4°C for 1 min, the specimen was always kept at certain humidity. Each section was blocked with 100ul of 10% goat serum at room temperature for 30 min and washed with 0.01 mol/L PBS (pH 7.4) three times. Rabbit anti-human IgG (H+L)-FITC antibodies were diluted at 1:200, and added to each section, and then kept away from light and incubated at 37°C for 30 min, and washed at 1 min each time for four times. Lastly, a drop of buffered glycerin was added to cover the glass slide. The images were collected by a laser confocal microscope at 488 nm.

### Transmission Electron Microscopy

The skin specimens were cut into small pieces and placed in half-strength Karnovsky fixative. Samples were fixed while on a rotator at room temperature for 2 h and then washed in PBS at 4°C. Postfixation was in osmium tetroxide for 2 h at a 4°C temperature. Specimens were dehydrated in a graded ethanol series (15 min each), stained *en bloc* in uranyl acetate, and embedded in Eponate 812 resin *via* propylene oxide. Semithin sections (0.5 mm) cut on an ULTRACUT UCT/UC6 ultramicrotome (Leica, UK) were stained with azure II and methylene blue. Ultrathin sections (80-90 nm) were stained with 50% alcoholic uranylacetate (15 min) and lead citrate (10 min) and examined by a JEM-1230 transmission electron microscope (Tokyo, Japan).

### TUNEL Assay

The experimental mouse skin samples were embedded in OCT. Cryopreserved tissue sections were fixed in 4% paraformaldehyde in PBS for 20 min and subjected to TUNEL assay using the *in situ* cell death detection kit (Roche) according to the manufacturer’s instructions. Following permeabilization and wash, sections were incubated with a reaction mixture containing TdT and fluorescence-conjugated dUTP for 1 h at 37°C. The labeled DNA was examined under a confocal microscope.

### Statistics

The t-test was performed for comparisons between two groups by SPSS (SPSS, Inc., Chicago, IL, USA). GraphPad Prism 5.0 was used to compare differences and drew a graph. *P* < 0.05 was considered statistically significant.

### Study Approval

Written informed consent was obtained from all patients and healthy donors. The studies, including the animal study, were performed according to the Declaration of Helsinki and were approved by the medical ethical committee of Peking University First Hospital.

## Results

### Expression and Purification of the Recombinant of DP-C in *E. coli*

**Figure 1:**
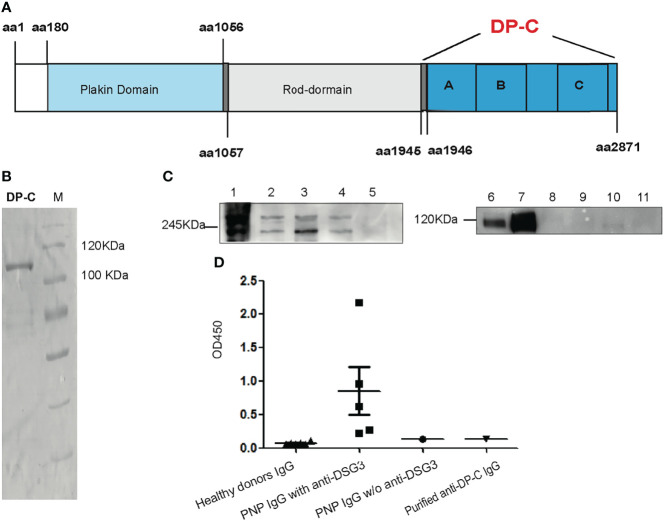
Recombinant DP-C and the specificity of the DP-C affinity purified IgG. **(A)** Scheme chart of desmoplakin. DP-C was started from aa1945 to aa2871 containing plakin repeat domains A, B, and C. **(B)** Recombinant protein DP-C on SDS-PAGE stained by Coomassie brilliant blue, and the molecular mass is about 106 kDa. **(C)** IP-IB assay confirmed the specificity of the DP-C affinity purified IgG. In the IP assay before the IB assay, the extract of HaCat cells was the substrate. The left panel C1-5 showed the IB process with commercial monoclonal antibodies against desmoplakin. C1: IP complex of another commercial anti-desmoplakin antibody; C2: total protein of HaCat as positive control; C3: the IP complex of DP-C affinity purified IgG; C4: the IP complex of N-terminus of desmoplakin affinity purified IgG; C5: the IP complex of healthy donor IgG. Bands of 250 kDa and 210 kDa were detected in the IP complex of DP-C purified IgG (C3) the same as C1, C2, and C4. The right panel C6-11 revealed in the IB process bands expected to be 130 kDa were detected by the monoclonal antibody against desmoglein 3. C6: total protein of HaCat cells as positive control; C7: IP complex of another commercial monoclonal anti-desmoglein 3 (DSG3) IgG; C8: IP complex of normal mouse IgG; C9: IP complex of DP-C affinity purified IgG; C10: IP complex of N-terminus of desmoplakin affinity purified IgG; C11: IP complex of healthy donor IgG. The band of Dsg3 was negative in the complex of DP-C purified IgG (C9), the same as the negative control (C11 and C10). **(D)** The OD450 value of a group of six healthy donors, five PNP with anti-desmoglein 3, one PNP without (w/o) anti-desmoglein 3, and purified anti-DP-C IgG was determined using an ELISA test of anti-Dsg3 IgG. The OD450 value of anti-DP-C was lower than the value of group of PNP IgG with anti-desmoglein 3, and equal to the PNP IgG w/o anti-demoglein 3, close to the healthy donors.

Recombinant DP-C from aa1945 to aa2781 of desmoplakin was expressed by *E. coli* BL21. It contained plakin A, B, and C domains (**Figure 1A**). Between the B and C domains was the Linker subdomain (not shown in **Figure 1A**). The mass weight turned out to be about 106 kDa. An isolated band was detected by Coomassie-staining on the SDS-PAGE after purification by the Ni-NTA column (**Figure 1B**).

### Purification of Autoantibodies Against DP-C from the Patient and the Specificity of the Antibodies

Total IgG was obtained from the PNP patient’s serum by rProteinA column (GE). Autoantibodies against DP-C were purified by the recombinant DP-C coupled CNBr column from the total IgG. Before the functional experiments, the specificity of the DP-C affinity purified IgG was confirmed by IP-IB with HaCat cells extract as substrate and ELISA (4). The commercial monoclonal antibodies against DP, the purified anti-DP-C IgG, N-terminus of desmoplakin affinity purified IgG, and IgG of the healthy donors were incubated with the extract of HaCat cells for IP, and then the IB assay was performed using another monoclonal antibodies against desmoplakin I/II. As shown in the left panel of **Figure 1**C, desmoplakin I and II (protein bands mass weighted 250 kDa and 210 kDa) were detected in the IP complex of DP-C affinity purified IgG (C3), the same as the IP complex of commercial monoclonal anti-desmoplakin antibody (C1), the extract of HaCat cells (C2), and the IP complex of N-terminus of desmoplakin affinity purified IgG (C4). To exclude the possibility of pathogenic interference of autoantibodies against the antigen of pemphigus vulgaris presented in our preparation, the IP-IB assay with the monoclonal antibodies against desmoglein 3 was also performed. As shown in the right panel of **Figure 1C**, desmoglein 3 was detected in the extract of HaCat cells (C6) and the IP complex of monoclonal antibodies against desmoglein 3 (C7) worked as the positive control. The complex of DP-C affinity IgG (C9) was negative, the same as the healthy donor control (C11). To further determine whether the DP-C affinity purified IgG can react with desmoglein 3 or not, ELISA was performed with IgG from PNP patients and healthy volunteers. Five PNP sera, all with antibodies against Dsg3, were performed as the positive controls. One PNP serum without antibodies to desmoglein 3 and six healthy adults served as controls. The cut-off OD value was 0.137 (Mean+3SD). The negative PNP and anti DP-C autoantibodies were both tested negative (both of their OD =0.135), while the mean OD value of the positive control is 0.272, 0.623, 2.175, 0.962, 0.221, respectively (**Figure 1D**).

### Detachment of Keratinocytes Induced by the Autoantibodies Against DP-C

**Figure 2:**
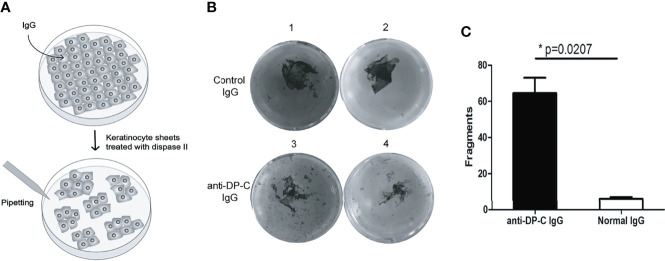
DP-C affinity purified IgG induces loss of keratinocyte adhesion. Keratinocyte monolayers were incubated with the DP-C IgG and IgG from healthy volunteers. **(A)** The scheme of keratinocyte dissociation assays. **(B)** B1 and 2 are the group of normal human IgG, while B3 and 4 are the group of anti-DP-C IgG. **(C)** The numbers of fragments induced by the anti-DP-C IgG were significantly more than the IgG of healthy donors. The numbers of fragments induced by the anti-DP-C IgG were significantly more than the IgG of healthy donors (*P <*0.05).

To examine the pathogenicity of the anti-DP-C-specific IgG *in vitro*, we performed a dispase-based keratinocytes dissociation assay with normal human epidermal keratinocytes (HEK) (**Figure 2A**). IgG of healthy adults were use as negative control. The numbers of dissociated fragments caused by anti-DP-C IgG were significantly higher than normal control (*P*-value=0.0207, **Figures 2** **B, C**).

### Pathogenesis of Autoantibodies Against DP-C in Mice

**Figure 3:**
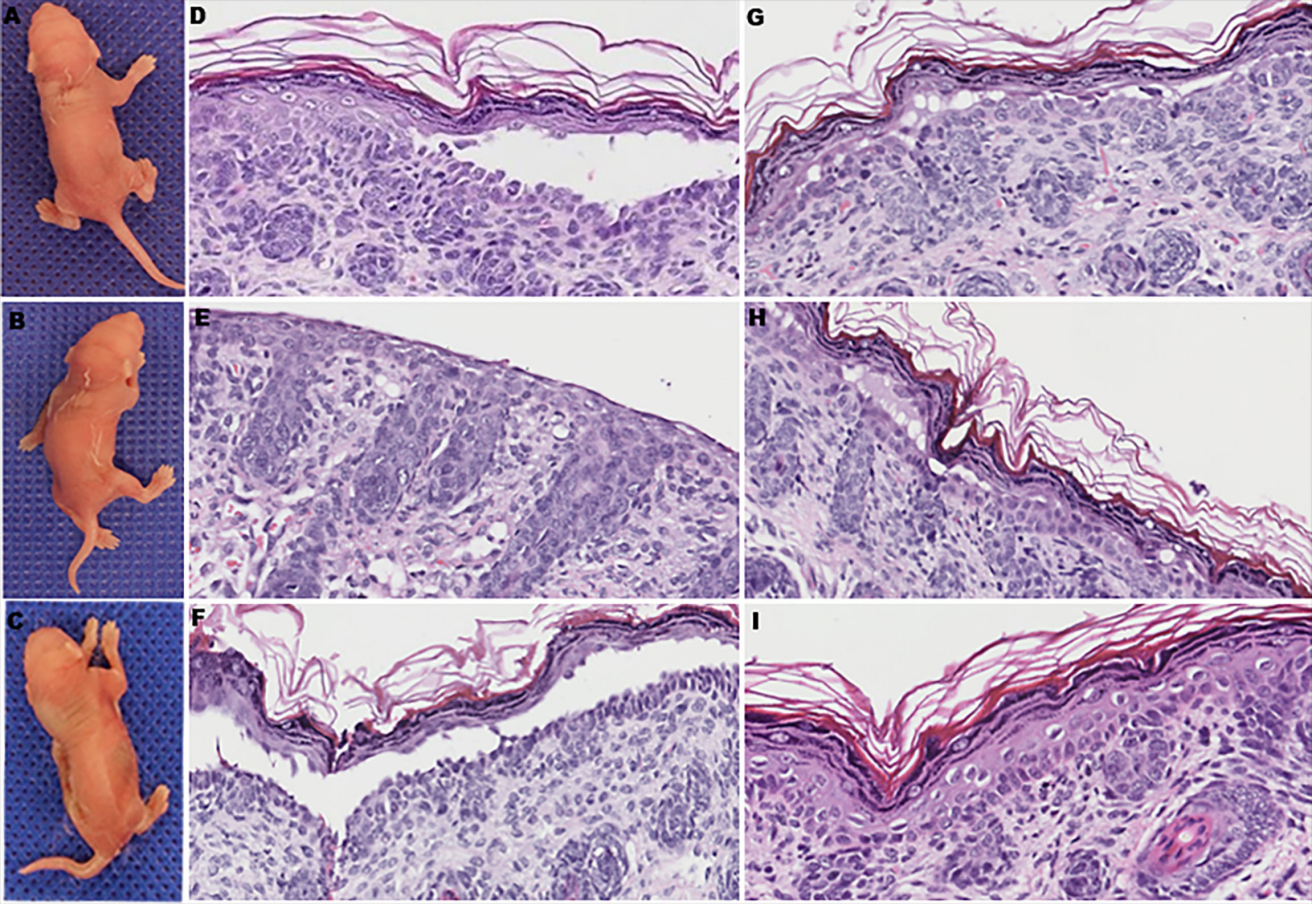
The clinical manifestations and pathological changes of skin in mice after being injected with anti-DP-C IgG and normal IgG. **(A, B)** Blisters on the backs of the neonatal mice injected with 3.3 mg/g of anti-DP-C IgG. **(C)** The skin of a newborn mouse accepted 3.3 mg/g normal human IgG was intact. **(D–G)** All of the mice injected with 3.3 mg/g of anti-DP-C IgG showed suprabasilar acantholysis in histology. Stratum corneum and granular layer absence was seen in **(E)** because of the strong degree of acantholysis.. **(H)** One of the mice injected with 1.7 mg/g of anti-DP-C showed suprabasilar acantholysis in histology. **(I)** Mice injected with 3.3 mg/g of normal human IgG showed no change in histology.

**Figure 4:**
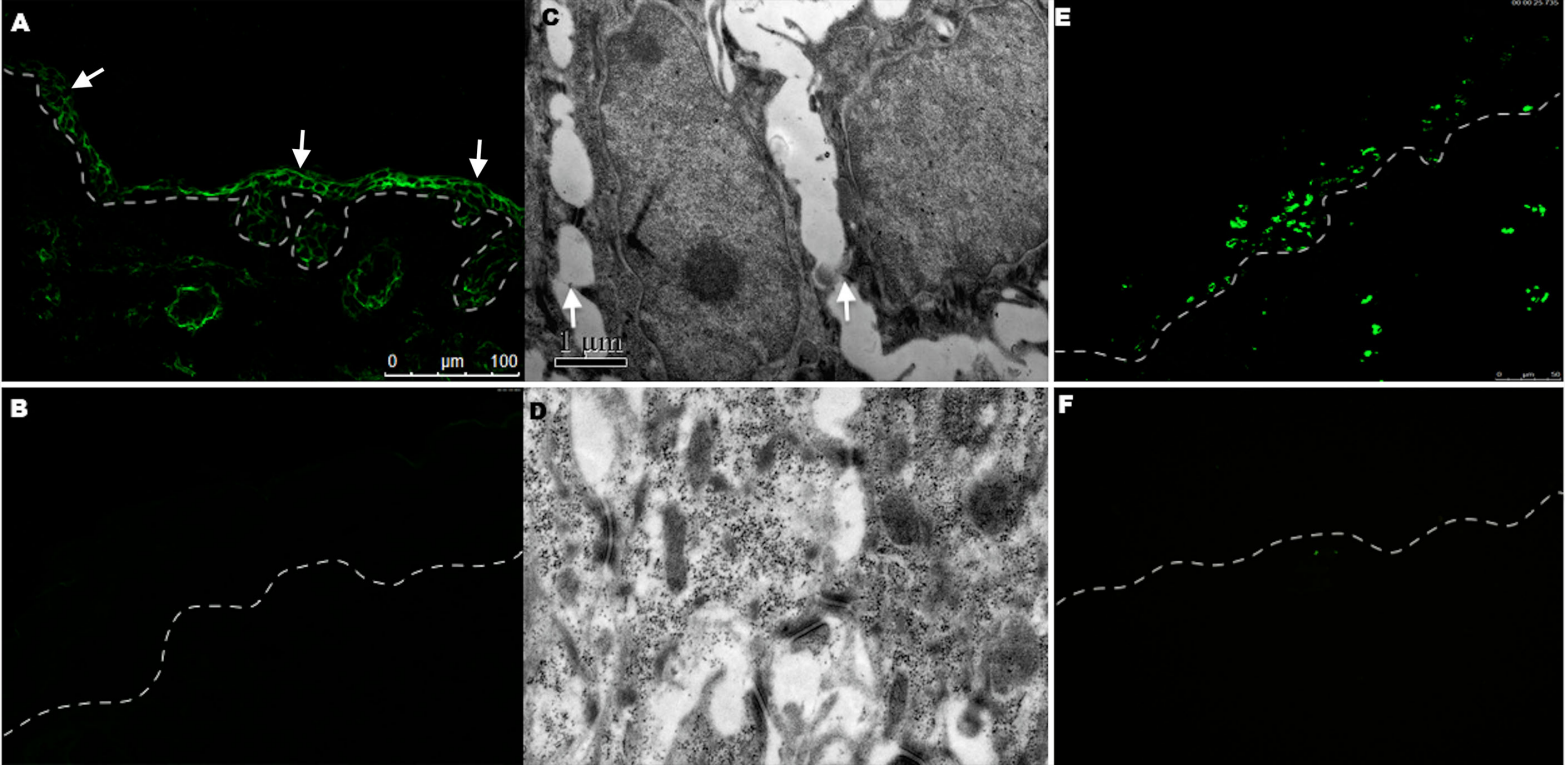
DIF, electronic microscopy examination, and TUNEL staining in the group after being injected with 3.3 mg/g anti-DP-C IgG and normal IgG. **(A)** Human IgG deposition was found on keratinocyte cell surfaces (arrows) in the epidermis of mice injected with anti-DP-C IgG by direct immunofluorescence. **(B)** No IgG was deposited in the normal IgG injected group. **(C, D)** The electronic microscopy examination showed that disconnection of keratin intermediate filaments from desmosomes (arrows) in the mice injected with anti-DP-C IgG **(C)** compared with the normal IgG injected group **(D)** (bar=1μm). **(E, F)** TUNEL staining on the skin of neonatal mice injected with anti-DP-C IgG or normal human IgG. Positive TUNEL labeling was present in the epidermis of the mice injected with anti-DP-C IgG **(E)**. (bar=50 μm)

To further determine whether anti-DP-C IgG in PNP sera was pathogenic *in vivo*, the purified autoantibodies were injected subcutaneously into 16 neonatal mice at different doses. Before the injection experiments, the titer of the original anti-DP-C IgG before injection was 1:640 tested by the IIF on rat bladder. The maximal dose was then set at 5 mg, the middle dose was set at 2.5 mg, and the minimal dose was set at 1 mg. The corresponding concentrations were about 3.3 mg/g, 1.7 mg/g, and 0.6 mg/g, respectively. The sera of the mice were all positive tested by IIF on the rat bladder, and the titers of the 3.3 mg/g dose group were 1:20 to 1:40, the titers of the 1.7 mg/g dose group were both 1:10, and the titers of the 0.6 mg/g group were 1:10, which were in the trend of dose-dependent. Normal human IgG was injected as a control at the same dose 24 h later. Obvious blisters on the back of neonatal mice accepting 3.3 mg/g anti-DP-C IgG can be seen (**Figures 3A, B**) but not the ones injected with normal human IgG (**Figure 3C**). One of the mice injected with 1.7 mg/g anti-DP-C IgG formed blisters on the back after two pinches. But 0.6 mg/g anti-DP-C antibodies didn’t induce visible blisters in neonatal mice even after being pinched three times. The mean pinch scores of different dose groups were calculated (**Table 1**). The highest dose group (3.3 mg/g) got the highest score, and the score of the lowest group (0.6 mg/g) and the healthy control group were zero, which is dose-dependent. Hematoxylin and eosin staining of skin biopsy showed intraepidermal blisters formation and significantly accelerated acantholysis in the spionous cell layer in the 3.3 mg/g group (**Figures 3D–G**). Acantholysis was found in one of the two mice injected with a lower concentration of IgG (1.7 mg/g (**Figure 3H**), 0.6 mg/g). In contrast, mice injected with 3.3 mg/g normal human IgG does did not show such histological changes (**Figure 3I**). In DIF, positive human IgG deposition was observed on the keratinocyte cell surfaces in the spinous layers of the biopsy skin 3 h after injection with 3.3 mg/g anti-DP-C IgG (**Figure 4A**). No deposition of IgG on the epidermis of normal control was found (**Figure 4B**).

**Table 1:** Acantholysis and mean pinch scores of the mice in the group A (anti-DP-C IgG) and group B (normal IgG) at different doses of 1-fold (3.3 mg/g), 1/2 fold (1.7 mg/g), and 1/5 fold (0.6 mg/g).

Group		Acantholysis(Pn/Tn)	Mean pinch score
Anti-DP-C IgG	1-fold	4/4	2.25
	½-fold	1/2	1
	**⅕** -fold	1/2	0
Normal control IgG	1-fold	0/4	0
	½-fold	0/2	0
	**⅕** -fold	0/2	0

Pn/Tn: number of positive/number of totals.

To further observe the changes causing acantholysis, the electronic microscopy examination was performed and showed the disconnection of keratin intermediate filaments from desmosomes (arrows) in the mice injected with anti-DP-C IgG (**Figure 4C**) compared with the control group (**Figure 4D**).

A TUNEL assay was performed to further investigate whether apoptosis occurred in the mice skin, and positivity was observed in the epidermis 24 h after anti-DP-C-IgG injection (**Figure 4E**), but not in the skin after being injected with normal human IgG (**Figure 4F**).

## Discussion

The true pathogenesis of PNP in its immunological part is still unclear. As deduced above, the low presence and levels of anti-desmogleins could not explain the whole story of PNP humoral responses. In this study, we demonstrated that the C-terminus of desmoplakin (DP-C) might be a pathogenic epitope in PNP. In our experiments, anti-DP-C autoantibodies caused detachment of keratinocytes *in vitro* dispase-dependent keratinocyte dissociation assay. Furthermore *in vivo*, by transferring IgG passively into the neonatal mice assay, we found that the DP-C autoantibodies caused blisters and acantholysis in mice skin. Moreover, the electronic microscopy examination showed the disconnection of keratin intermediate inflaments from desmosomes, similar to the phenomenon revealed by perinuclear retraction of intermediate filaments that had disconnected from the inner dense plaque of desmosomes in heredity epidermolysis bullosa caused by genetic truncation of the DP tail (10). Because the subdomains “A”(aa1960-aa2208), “B”(aa2244-aa2446), “C”(aa2609-aa2822)shown in **Figure 1A** were in the C-terminus interacting with intermediate inflaments (13), the polyclonal antibodies IgG might recognize the DP-C domain, then destroy the connection of intermediate inflaments to the DP, causing the unsteady structure of the desmosome plaque. By detecting apoptosis of keratinocytes in the TUNEL assay in the skin of neonatal mice after injection of the anti-C terminus of desmoplakin autoantibodies, we demonstrated that the anti-DP-C IgG triggered DNA fragmentation of lesional epidermal cells in neonatal mice. The apoptotic DNA fragments were presented with acantholysis and induced by anti-DP-C IgG, which indicated that the apoptosis of kerationocytes could be induced not only by cellular immunity (14) but also humoral immunity to the desmoplakin.

Nousari et al. proved that multiple autoantibodies against the homologous tail region of the plakin family in neonatal mice were pathogenic in PNP by passively transferring purified IgG into neonatal mice. This specific IgG crude reacted roughly to envoplakin, moderately to periplakin and plectin, and weakly to desmoplakin I and BPAG1. Twelve hours later, mucutanous blisters were present and all epithelial surfaces were acantholytic and deposited with human IgG (15). This study suggested that autoantibodies against the plakin family could cause acantholysis. However, the quantity and the affinity-purifed autoantibodies against the desmoplakin I were little and weak. There’s one possibility that the conformational interaction of DP and its autoantibodies were overlooked at that time. Since then, the concept of plakin antibodies in the pathogenesis of PNP has been overthrown by the findings of acantholysis caused by desmoglein antibodies from PNP (4).

In many points of view, antibodies against the plakin family were incapable of causing pathological change due to their intracellular localization. However, BPAG1 has been shown to be pathogenic in subepidermal blister formation as a bullous pemphigoid antigen, and Kiss et al. proposed an alternative idea that autoantibodies could penetrate living cells and altered their function (16). Karla Cauza et al. proved that the autoantibodies against DP (purified by the peptide GNSSYSYSYSSFS) were bound at the cell surface of cultured human keratinocytes, internalized *via* plasmalemmal vesicles, and were consecutively within tubulovesicular structures inside the cells (17). Anna Zakrzewicz et al. has proven that the FcRn may play a direct role in the pathogenesis and transport of autoantibodies in pemphigus (18). In our previous study, the purified specific anti-EPL and anti-PPL autoantibodies from PNP sera could enter the living keratinocytes by internalization and dissociate cultured confluent keratinocytes (19). Besides, Cauza *et al.* found that anti-desmoplakin autoantibodies of erythema multiform patients could be internalize into keratinocytes by plasmalemmal vesicles *in vitro* (17). Our results further demonstrate the pathogenic role of anti-DP-C antibodies *in vivo*, though this mechanism needs to be further investigated.

Desmoplakin, as a component of functional desmosomes for cytoskeletal structure and membrane attachments, has been shown to be critical in the stability of intercellular adhesion (20). In addition, the pathogenic mechanism of anti-DP-C autoantibodies in PNP might be inferred from some heredity diseases. Known as “lethal acantholytic epidermolysis bullousa”, named by Mcgrath et al., reported a neonate with genetic truncation of DP tail, more than two-thirds of whose skin was denuded. Light microscopy showed hyperkeratosis and keratinocyte cell-cell separation with suprabasal clefting, and electron microscopy revealed that there was complete detachment from the intermediate filament network from the desmosomal plaques (21). In our study, detachment of intermediate filament was also observed by electron microscope, from which we speculated that the anti-DP-C autoantibodies might bind the C-terminal of DP and then destroy the association between DP and keratin filament. Therefore, the pathological roles of anti-Dsg3 autoantibodies and anti-DP-C autoantibodies might be different.

Some authors reported that antibodies against desmoplakin were occasionally found in pemphigus vulgaris, but it turned out that these antibodies could be found by IB (22–24). They preferred to assume that the epitope-spreading phenomenon induced the antibodies against desmoplakin. Even though PNP was thought to be a humoral- and cellular-mediated disease in which epitope spreading participates (25). Autoantibodies against desmoplakin were still mostly detected by IP-IB in our diagnostic assays and in the literature. For that reason, we inferred that the roles of antibodies against desmoplakin were truly different in PNP and PV, since IP-IB preserved the conformational interactions of DP and its autoantibodies.

Clinically, PNP patients whose desmoglein 3 levels were negative also had autoantibodies against DP. Our study suggested that autoantibodies against desmoplakin were another explanation for blister formation and acantholysis. Together, the autoantibodies against both desmoplakin and desmoglein 3 induce the different phenotypes of pemphigus vulgaris and PNP in the aspect of humoral immunity. After all, our findings can be a complement to the acantholysis in those pemphigus patients who didn’t have autoantibodies against desmoglein 3.

Our study was limited by the number of patients who underwent plasmapheresis, since it was no longer a common treating strategy for PNP in recent decades. It was also undeniable that the role of desmoplakin in this model was challenged by other plakins sharing similar plakin repeat domains and linker subdomains. Still, our previous study failed in observing any phenotype in mouse skin to prove the pathogenesis of antibodies against envoplakin and periplakin. In the future, the rabbit polyclonal antibodies against the C-terminus of desmoplakin could be used to furtherly prove this hypothesis.

We believe that, taken together, these results are the first to describe desmoplakin as a pathogenic antigen in PNP. The autoantibodies against DP-C might contribute to the development of this rare disorder in both animal and human keratinocyte models, along with the participation of desmoglein 3 autoantibodies and its specific T cells. More investigations are needed to reveal the mechanisms behind the phenomena.

## Data Availability Statement

The raw data supporting the conclusions of this article will be made available by the authors, without undue reservation.

## Ethics Statement

The studies involving human participants were reviewed and approved by the Medical Ethical Committee of Peking University First Hospital. Written informed consent to participate in this study was provided by the participants’ legal guardian/next of kin. The animal study was reviewed and approved by the Medical Ethical Committee of Peking University First Hospital.

## Author Contributions

MW, ZL, DB, XC, and XZ contributed to the design of the study. XW, RW, LW, YZ, YC, and CZ performed the experiments. MW revised the manuscript. All authors contributed to the article and approved the submitted version.

## Funding

This study was supported by the National Natural Science Foundation of China (grant numbers: 81130030 and 81000694) and the Beijing Natural Science Foundation (7172214).

## Conflict of Interest

The authors declare that the research was conducted in the absence of any commercial or financial relationships that could be construed as a potential conflict of interest.

## Publisher’s Note

All claims expressed in this article are solely those of the authors and do not necessarily represent those of their affiliated organizations, or those of the publisher, the editors and the reviewers. Any product that may be evaluated in this article, or claim that may be made by its manufacturer, is not guaranteed or endorsed by the publisher.
